# Biological Activities of Plant Food Components: Implications in Human Health

**DOI:** 10.3390/foods10020456

**Published:** 2021-02-19

**Authors:** Carla Gentile

**Affiliations:** Department of Biological, Chemical and Pharmaceutical Sciences and Technologies (STEBICEF), University of Palermo, Viale delle Scienze, 90128 Palermo, Italy; carla.gentile@unipa.it; Tel.: +39-091-23897423

Scientific data and epidemiological evidence collected over the last fifty years have shown that nutrition plays a decisive role in human health. While before it was considered essentially a non-disease condition, health is nowadays felt as a state of complete physical, mental, and social wellness, according to the definition of the World Health Organization. Eating properly is not only necessary to meet energy needs, avoiding syndromes associated with nutritional deficiency and excess, but it actively contributes to improve human health. The functional meaning of nutrition as surely protective and possibly therapeutic is today amply demonstrated by scientific evidence. Additionally, the increasing sensitivity to the healthy role of nutrition makes consumers more and more careful when choosing high quality foods.

The functional role of nutrition is due to specific small molecules with biological activity. These dietary compounds neither act as energy substrates or plastic materials for cells, nor as enzymatic cofactors. However, due to their peculiar bioactivity, they can benefit human health.

Plants are the most important source of bioactive molecules and still represent the main resource in the quest for new drugs. The interest in dietary phytochemicals is justified by the results from numerous epidemiological studies that demonstrate how diets rich in plant foods are able to prevent several human diseases, including cardiovascular, neoplastic, neurodegenerative, and metabolic pathologies. Of course, it is not possible to demonstrate that the positive effects resulting from the intake of a specific plant food are due to a particular component, for the presence of other phytochemicals and any synergistic effects cannot be neglected. However, experimental results showing biological activity for many pure dietary phytochemicals suggest that particular components may participate in the protective effects associated with the consumption of the plant food it can be sourced from.

The documented biological activity of phytochemicals is expressed through several protective effects, such as antioxidant, anti-inflammatory, antimicrobial, antitumor, immunomodulatory, neuroprotective, antihypertensive, antidiabetic actions. The commercial success of many plant-based supplements is based on these proven biological effects.

Although the protective effect associated with the consumption of plant foods is ascribable to a number of mechanisms, the biological activity of these phytocomponents has frequently been related to their ability to function as antioxidants. Many phytochemicals are in fact redox-active molecules and thus are able to influence the cellular redox balance. For this reason, besides protecting cells from oxidative stress phenomena, phytochemicals influence numerous redox-sensitive biological targets that regulate several important cellular functions. Additionally, the activity of phytochemicals can also involve interactions of these small molecules with various biological targets, including proteins, DNA and lipids, and consequently a possible alteration of their function [1].

This Special Issue addresses the biological activity of dietary phytochemicals, either purified [2] or in extracts from plant foods, and speculates on their potential effects on human health. The studied plant foods include edible parts (fruits, seeds, leaves, and flowers) of plants common in the Mediterranean area, such as pomegranate (*Punica granatum*) [3], oregano (*Origanum vulgare)* [4], and purslane (*Portulaca olearacea*) [5], but also of several tropical plants, including annona (*Annona cherymola*) [6], herba mate (*Ilex paraguariensis*), guaranà (*Paullinia cupana*) [7], and ash gourd (*Benincasa hispida*) [8]. Bioactivity was studied in terms of radical scavenging and antioxidant [5,6,7,9], antimicrobial [10], and antiproliferative activity [4].

The collected results suggest that the intake of the aforementioned plant foods in the context of a balanced diet could be beneficial to health. In addition, considering the high concentration of bioactive molecules in some of the observed plant matrices, the presented results hint at the possibility of using some of the studied plant extracts in food functionalization or in the formulation of dietary supplements.

Due to their ability to exert several biological effects that are potentially useful for human health, over the years, dietary phytochemicals have drawn increasing interest in human nutrition research.

The papers collected in this Special Issue contribute to the growth of this research area.

I would like to thank all the authors and the reviewers of the papers published in this Special Issue for their great contributions and effort. I am also grateful to the editorial board members and to the staff of the journal for their kind support during the preparation of this Special Issue.

## References

[1] Caradonna F., Consiglio O., Luparello C., Gentile C. (2020). Science and Healthy Meals in the World: Nutritional Epigenomics and Nutrigenetics of the Mediterranean Diet. Nutrients.

[2] Meng X., Zhou J., Zhao C.-N., Gan R.-Y., Li H.-B. (2020). Health benefits and molecular mechanisms of resveratrol: A narrative review. Foods.

[3] Di Stefano V., Scandurra S., Pagliaro A., Di Martino V., Melilli M.G. (2020). Effect of Sunlight Exposure on Anthocyanin and Non-Anthocyanin Phenolic Levels in Pomegranate Juices by High Resolution Mass Spectrometry Approach. Foods.

[4] Nanni V., Di Marco G., Sacchetti G., Canini A., Gismondi A. (2020). Oregano Phytocomplex Induces Programmed Cell Death in Melanoma Lines via Mitochondria and DNA Damage. Foods.

[5] Melilli M.G., Di Stefano V., Sciacca F., Pagliaro A., Bognanni R., Scandurra S., Virzì N., Gentile C., Palumbo M. (2020). Improvement of fatty acid profile in durum wheat breads supplemented with portulaca oleracea L. quality traits of purslane-fortified bread. Foods.

[6] Gentile C., Mannino G., Palazzolo E., Gianguzzi G., Perrone A., Serio G., Farina V. (2021). Pomological, Sensorial, Nutritional and Nutraceutical Profile of Seven Cultivars of Cherimoya (Annona cherimola Mill). Foods.

[7] Dabulici C.M., Sârbu I., Vamanu E. (2020). The bioactive potential of functional products and bioavailability of phenolic compounds. Foods.

[8] Sultana R., Alashi A.M., Islam K., Saifullah M., Haque C.E., Aluko R.E. (2020). Inhibitory Activities of Polyphenolic Extracts of Bangladeshi Vegetables against α-Amylase, α-Glucosidase, Pancreatic Lipase, Renin, and Angiotensin-Converting Enzyme. Foods.

[9] Taniguchi A., Kyogoku N., Kimura H., Kondo T., Nagao K., Kobayashi R. (2020). Antioxidant Capacity of Tempura Deep-Fried Products Prepared Using Barley, Buckwheat, and Job’s Tears Flours. Foods.

[10] Tamfu A.N., Ceylan O., Kucukaydin S., Ozturk M., Duru M.E., Dinica R.M. (2020). Antibiofilm and Enzyme Inhibitory Potentials of Two Annonaceous Food Spices, African Pepper (Xylopia aethiopica) and African Nutmeg (Monodora myristica). Foods.

